# Studying Musical and Linguistic Prediction in Comparable Ways: The Melodic Cloze Probability Method

**DOI:** 10.3389/fpsyg.2015.01718

**Published:** 2015-11-12

**Authors:** Allison R. Fogel, Jason C. Rosenberg, Frank M. Lehman, Gina R. Kuperberg, Aniruddh D. Patel

**Affiliations:** ^1^Department of Psychology, Tufts University, MedfordMA, USA; ^2^Department of Arts and Humanities, Yale-NUS CollegeSingapore, Singapore; ^3^Department of Music, Tufts University, MedfordMA, USA; ^4^MGH/HST Athinoula A. Martinos Center for Biomedical Imaging, CharlestownMA, USA; ^5^Department of Psychiatry, Massachusetts General Hospital, CharlestownMA, USA

**Keywords:** music, language, prediction, music cognition, melodic expectation, cloze probability

## Abstract

Prediction or expectancy is thought to play an important role in both music and language processing. However, prediction is currently studied independently in the two domains, limiting research on relations between predictive mechanisms in music and language. One limitation is a difference in how expectancy is quantified. In language, expectancy is typically measured using the cloze probability task, in which listeners are asked to complete a sentence fragment with the first word that comes to mind. In contrast, previous production-based studies of melodic expectancy have asked participants to sing continuations following only one to two notes. We have developed a melodic cloze probability task in which listeners are presented with the beginning of a novel tonal melody (5–9 notes) and are asked to sing the note they expect to come next. Half of the melodies had an underlying harmonic structure designed to constrain expectations for the next note, based on an implied authentic cadence (AC) within the melody. Each such ‘authentic cadence’ melody was matched to a ‘non-cadential’ (NC) melody matched in terms of length, rhythm and melodic contour, but differing in implied harmonic structure. Participants showed much greater consistency in the notes sung following AC vs. NC melodies on average. However, significant variation in degree of consistency was observed within both AC and NC melodies. Analysis of individual melodies suggests that pitch prediction in tonal melodies depends on the interplay of local factors just prior to the target note (e.g., local pitch interval patterns) and larger-scale structural relationships (e.g., melodic patterns and implied harmonic structure). We illustrate how the melodic cloze method can be used to test a computational model of melodic expectation. Future uses for the method include exploring the interplay of different factors shaping melodic expectation, and designing experiments that compare the cognitive mechanisms of prediction in music and language.

## Introduction

Recent years have seen growing interest in cognitive and neural relations between music and language. Although there are clear differences between the two— for example, language can convey specific semantic concepts and propositions in a way that instrumental music cannot (Slevc and Patel, 2011) — they share several features. For example, both language and music involve the generation and comprehension of complex, hierarchically structured sequences made from discrete elements combined in principled ways (Patel, 2003; Koelsch et al., 2013), and both rely heavily on implicit learning during development (Tillmann et al., 2000).

While neuropsychology has provided clear cases of selective deficits in linguistic or musical processing following brain damage (e.g., Peretz, 1993), several neuroimaging studies of healthy individuals suggest overlap in the brain mechanisms involved in processing linguistic and musical structure. One early demonstration of this overlap came from event-related potential (ERP) research, which revealed that a component known as the P600 is observed in response to syntactically challenging or anomalous events in both domains (Patel et al., 1998). Later research using MEG and fMRI provided further suggestions of neural overlap in structural processing, e.g., by implicating Broca’s region in the processing of tonal-harmonic structure (e.g., Maess et al., 2001; Tillmann et al., 2003; LaCroix et al., 2015; Musso et al., 2015; though see Fedorenko et al., 2012). To resolve the apparent contradiction between evidence from neuropsychology and neuroimaging, Patel (2003) proposed the shared syntactic integration resource hypothesis (SSIRH). The SSIRH posits a distinction between domain-specific representations in long-term memory (e.g., stored knowledge of words and their syntactic features, and of chords and their harmonic features), which can be separately damaged, and shared neural resources which act upon these representations as part of structural processing. This “dual-system” model proposes that syntactic integration of incoming elements in language and music involves the interaction (via long-distance neural connections) of shared “resource networks” and domain-specific “representation networks” (see Patel, 2013 for a detailed discussion, including relations between the SSIRH and Hagoort’s (2005) “memory, unification, and control” model of language processing).

The SSIRH predicted that simultaneous demands on linguistic and musical structural integration should produce interference. This prediction has been supported by behavioral and neural research (for a review, see Kunert and Slevc, 2015). For example, behavioral studies by Fedorenko et al. (2009) and Slevc et al. (2009) have shown that it is particularly difficult for participants to process complex syntactic structures in both language and music simultaneously (see also Hoch et al., 2011; Carrus et al., 2013; though cf. Perruchet and Poulin-Charronnat, 2013). Additionally, Koelsch et al. (2005) conducted an ERP study that observed an interaction between structural processing in language and music, as reflected by effects of music processing on the left anterior negativity (LAN, associated with processing syntax in language) and effects of language processing on the early right anterior negativity (ERAN, associated with processing musical syntax).

In addition to structural integration, it has been suggested that prediction may be another process that operates similarly in language and music (Koelsch, 2012a,b; Patel, 2012). Prediction is increasingly thought to be a fundamental aspect of human cognition (Clark, 2013), and is a growing topic of research in psycholinguistics (Van Petten and Luka, 2012; see Kuperberg and Jaeger, in press for a recent review). It has become clear that we regularly use context to predict upcoming words when comprehending language (Tanenhaus et al., 1995; Altmann and Kamide, 1999; Wicha et al., 2004; DeLong et al., 2005). This has been demonstrated using ERPs, a brain measure with millisecond-level temporal resolution that allows one to study cognitive processing during language comprehension. Recent evidence from ERP research has suggested that prediction in language processing occurs at multiple distinguishable levels (e.g., syntactic, semantic, phonological) (Pickering and Garrod, 2007; Kuperberg and Jaeger, in press).

Strong lexical predictions for a specific word occur when multiple types of information within a linguistic context constrain strongly for the semantic features, the syntactic properties, and the phonological form of a specific word. For example, the sentence *“The piano is out of ____”* leads to a strong expectation for the word “tune”, so one can refer to this as a high lexical constraint sentence. It is well established that unexpected words following these contexts evoke a larger N400 ERP component (occurring 300–500 ms after the presentation of the final word) than expected words (Kutas and Hillyard, 1980; Kutas and Hillyard, 1984; Kutas and Federmeier, 2011). Such unexpected words do not necessarily need to be anomalous to produce an N400: predictions can also be violated with words that are perfectly coherent and non-anomalous. For example, if the final word delivered in the above sentence is “place” (i.e., “*The piano is out of place*”) this word still violates a lexical prediction for the highly expected word “tune.” As in the previous example, the N400 elicited by “place” would be larger than that elicited by “tune,” as it is less expected. Moreover, in recent ERP research, violations of specific lexical predictions with other plausible words have also been observed to elicit a late anteriorly distributed positive component. This late frontal positivity has been observed at various time points after the N400, often peaking around 500–900 ms after the presentation of a critical item (Federmeier et al., 2007; Van Petten and Luka, 2012). Importantly, unlike the N400, the late frontal positivity is not produced by words that follow non-constraining contexts, when comprehenders have no strong prediction for a particular word (e.g., “place” following the context, “After a while, the boy saw the...”).

Predictions in language are not always at the level of specific lexical items: they can also be generated at the level of semantic-syntactic statistical contingencies that determine the structure of an event (‘who does what to whom’) (Kuperberg, 2013). For example, at a certain point in a sentence we might expect a certain syntactic category of word, like a noun-phrase, with certain coarse conceptual features, such as animacy. For example, in the sentence *“Mary went outside to talk to the ____”* there is no strong indication of which word will come next, but it is clear that it must be an animate noun-phrase (Mary would likely not talk to an inanimate object like a truck). Violations of these semantic-syntactic structural predictions have been observed to elicit a different neural response from the anterior positivity discussed above, namely the P600 (a late posterior positivity, peaking from around 600 ms after onset of the violating word; see Kuperberg, 2007 for a review). This provides evidence that distinct neural signatures may be associated with violations of strong predictions at different representational levels (e.g., a late anterior positivity evoked by violations of strong lexical predictions, Federmeier et al., 2007; a late posterior positivity evoked by violations of strong semantic-syntactic predictions, Kuperberg, 2007; see Kuperberg, 2013 for discussion). The functional significance of these late positivites (both frontal and posterior) evoked by strong prediction violations remains unclear. One possibility, however, is that they reflect the neural consequences of suppressing the predicted (but not presented) information and adapting one’s internal representation of context in order to generate more accurate predictions in the future (e.g., see Kuperberg, 2013; Kuperberg and Jaeger, in press, for discussion).

Turning to music, expectation has long been a major theme of music cognition research. Meyer (1956) first suggested a strong connection between the thwarting of musical expectations and the arousal of emotion in listeners. In recent years, theories of musical expectation have been brought into a modern cognitive science framework (e.g., Margulis, 2005; Huron, 2006; Huron and Margulis, 2010; Pearce et al., 2010), and expectation has been studied empirically with both behavioral and neural methods (e.g., Steinbeis et al., 2006). It is increasingly recognized that multiple sub-processes are involved in musical expectation (see Huron, 2006, for one theoretical treatment). Empirical research has shown that predictions are generated for multiple aspects of music, such as harmony, rhythm, timbre, and meter (Rohrmeier and Koelsch, 2012). Such expectations are thought to be automatically generated by enculturated listeners (Koelsch et al., 2000; Koelsch, 2012a).

Here, we focus on melodic prediction, and specifically on expectations for upcoming notes in monophonic (single-voice) melodies based on implicit knowledge of the melodic and harmonic structures of Western tonal music (Tillmann et al., 2000). For those interested in relations between predictive mechanisms in music and language, melodic expectancy provides an interesting analog to linguistic expectancy in sentence processing. Like sentences, monophonic melodies consist of a single series of events created by combining perceptually discrete elements in principled ways to create hierarchically structured sequences (Jackendoff and Lerdahl, 2006). Sentences and melodies have regularities at multiple levels, including local relations between neighboring elements and larger-scale patterns, e.g., due to underlying linguistic-grammatical or tonal structure.

In order to study relations between the cognitive mechanisms of prediction in sentences and melodies, it is necessary to measure prediction in these two types of sequences in comparable ways. In sentence processing, lexical expectancy has typically been measured using the cloze probability task, in which participants are asked to complete a sentence fragment with the first word that comes to mind (Taylor, 1953). For a given context, the percentage of participants providing a given continuation is taken as the “cloze probability” of that response. The cloze probability of an item is therefore a straightforward measure of how expected or probable it is. In addition to measuring the cloze probability of a particular word in relation to its context, it is also possible to use the cloze task to measure the ‘lexical constraint’ of a particular context by calculating the proportion of participants who produce a given word (see Federmeier et al., 2007). For example, a sentence such as *“The day was breezy so the boy went outside to fly a …”* would likely elicit the highly expected continuation *“kite”* from most participants, and thus be a ‘strongly lexically constraining’ context. In contrast, a sentence such as *“Carol always wished that she’d had a …”* would elicit a more varied set of responses, and thus be a ‘weakly lexically constraining’ context.

While expectancy in music has been measured in various ways over the years, to date there has been nothing comparable to the standard cloze probability method in language, i.e., a production-based task in which a person is presented with the beginning of a short coherent sequence and then asked to produce the event she thinks comes next. ^1^ Most behavioral studies of expectancy in music have used perceptual paradigms, such as harmonic priming paradigms or ratings of how well a tone continues an initial melodic fragment. Harmonic priming paradigms consist of a prime context followed by a target event, in which the degree of tonal relatedness between the two is manipulated. Typically, harmonically related targets are processed faster and more accurately than unrelated targets (Tillmann et al., 2014). These studies have shown that chords that are more harmonically related to the preceding context are easier to process, while there is a cost of processing chords that are less related or unrelated to the context (Tillmann et al., 2003). Another genre of priming studies has shown that timbre identification is improved when a pitch is close in frequency to the preceding pitch and harmonically congruent with the preceding context (Margulis and Levine, 2006). In studies using explicit ratings of expectancy, listeners are asked to rate how well a target note continues a melodic opening, e.g., on a scale of 1 (very bad continuation) to 7 (very good continuation) (e.g., Schellenberg, 1996). More recently, a betting paradigm has been used in which participants place bets on a set of possible continuations for a musical passage, and bets can be distributed across multiple possible outcomes (Huron, 2006). The betting paradigm has the advantage of providing a measure of the *strength* of an expectation for a specific item. However, like the “continuation rating” task, this task requires *post hoc* judgments, and is therefore not an online measure of participants’ real-time expectations. ERPs and measures of neural oscillatory activity can provide online measures of expectation in musical sequences (e.g., Pearce et al., 2010; Fujioka et al., 2012), but such studies have focused on perception, not production.

A handful of studies have used production tasks to measure musical expectancy, but they differ in important ways from the standard linguistic cloze probability task. Some studies have used extremely short contexts, in which participants are asked to sing a continuation after hearing only a single two-note interval, or even a single note (Carlsen, 1981; Unyk and Carlsen, 1987; Povel, 1996; Thompson et al., 1997; Schellenberg et al., 2002). Lake (1987) presented two-note intervals after establishing a tonal context consisting of major chords and a musical scale. However, no prior singing-based study of melodic expectation has used coherent melodies as the context (some studies using piano performance have used very long contexts, in which pianists have been asked to improvise extended continuations for entire piano passages, Schmuckler, 1989, 1990). Also, in all of these studies (and unlike in the linguistic cloze probability task), participants were asked to produce continuations of whatever length they chose in response to brief stimuli. The closest analog to a musical cloze task comes from a study of implicit memory for melody, in which listeners first heard a set of novel tonal melodies and then heard melodic stems of several notes and were asked to “sing the note that they thought would come next musically” (Warker and Halpern, 2005). However, the structure of the melodic stems was not manipulated, and the focus of the study was on implicit memory, not on expectation.

In order to advance the comparative study of prediction in language and music, it is necessary to develop comparable methods for studying prediction in the two domains. To this end, we have developed a melodic cloze probability task. In this task, participants are played short melodic openings drawn from novel coherent tonal melodies, and are asked to sing a single-note continuation. In an attempt to manipulate the predictive constraint of the melodies, the underlying harmonic structure of each opening (henceforth, ‘melodic stem’) was designed to either lead to a strong expectancy for a particular note, or not (see Materials and Methods for details). For each melodic stem, the cloze probability of a given note is calculated as the percentage of participants producing that note. The predictive constraint of a melodic stem is determined by examining the degree of agreement between participants’ responses. For example, if all participants sing the same note after a particular stem, the stem has 100% constraint. On the other hand, if the most commonly sung note is produced by 40% of the participants, then the stem has 40% constraint.

The melodic cloze probability method allows the cloze probabilities of notes to be quantitatively measured, and thus provides a novel way to study how different structural factors (e.g., local melodic interval patterns vs. larger-scale harmonic structure) interact in shaping melodic expectation. As demonstrated below, the method can also be used to test quantitative models of melodic expectation, such as Narmour’s (1990) “Implication-Realization” model, using naturalistic musical materials. In the future, the method can facilitate the design of studies comparing predictive mechanisms in language and music, e.g., by systematically manipulating constraint and cloze probabilities across linguistic and musical stimuli in behavioral or ERP studies of expectancy (cf. Tillmann and Bigand, 2015).

## Materials and Methods

### Participants

Fifty participants (29 female, 21 male, age range 18–25 years, mean age 20.3 years) took part in the experiment and were included in the data analysis (eight further participants were excluded due to difficulties with singing on pitch; see “Data Analysis”). All participants were self-identified musicians with no hearing impairment who had a minimum of 5 years of musical experience within the past 10 years (playing an instrument, singing, or musical training); 22 (44%) reported “voice” as one of their instruments. Participants had received a mean of 9.0 years of formal musical training on Western musical instruments (*SD* = 4.8) and reported no significant exposure to non-Western music. Participants were compensated for their participation and provided informed consent in accordance with the procedures of the Institutional Review Board of Tufts University.

### Materials

The stimuli consisted of 45 pairs of short novel tonal melodies created by the second author (JCR), a professional composer. Stimuli were truncated in the middle, creating “melodic stems.” The melodies ranged across all 12 major keys and employed variety of meters (3/4, 4/4, and 6/8 time signatures). Each stem was 5–9 notes long (*M* = 8.38 notes, *SD* = 0.83), and was played at a tempo of 120 beats per minute (bpm). Note durations varied from eighth notes (250 ms) to half notes (1000 ms). Stems contained no rests, articulation indications, dynamic variability, or non-diatonic pitches. All stimuli were created using Finale software with sampled grand piano sounds. Across all melodies, the highest and lowest pitch were A5 (880.0 Hz) and D3 (146.8 Hz), respectively, and the mean pitch was near E4 (329.6 Hz). On average, stems had a pitch range of 11.4 semitones (distance between the highest and lowest pitch in the stem, *SD* = 3.2 st). Male participants heard the melodic stems transposed down one octave. The average stem duration was 5.02 s (*SD* = 1.23).

**FIGURE 1:**
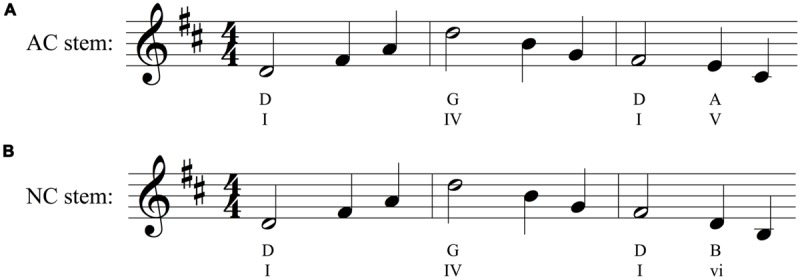
**(A)** Authentic cadence (AC) and **(B)** Non-cadence (NC) versions of one melodic pair (see text for explanation). The figure shows the AC and NC stems in Western music notation. Shown beneath each stem is a possible interpretation of the underlying implied harmonic progression (e.g., D, G, D, A chords in the AC stem), and harmonic functions (I, or tonic chord; IV, or subdominant chord; V, or dominant chord; vi or submediant chord). The stems of a pair are identical in length, key, rhythm, and melodic contour, and each consists of a single stream of notes with no accompaniment. In this pair the stems differ only in the identity of the final two notes, which are slightly lower in the second stem. Crucially, this small physical change alters the underlying harmonic progression.

Each stimulus pair consisted of two stems in the same musical key: one was an “authentic cadence” version, which was designed to create a strong expectation for a particular note, and the other was a “non-cadence” (NC) version, which was designed to *not* generate a strong expectation for a particular note. AC stems ended preceding a strong beat within the meter on the 2nd, 5th, or 7th scale degree and with an implied AC that would typically be expected to resolve to a tonic function. NC stems ended with an implied IV, iv, or ii harmony, with the last presented note never on the 2nd or 7th scale degree and rarely on the 5th. The two stems in each pair were identical in length, rhythm, and melodic contour; they differed only in the pitch of some of their notes, which influenced their underlying harmonic structure (see **Figure 1** for an example). On average, the two stems of an AC-NC melodic pair differed in 48.3% of their notes (*SD* = 28.5%). When notes of an AC-NC pair differed, they remained close in overall pitch height, on average 1.90 semitones apart (*SD* = 0.38).

The extent to which the two groups of stems projected a sense of key was compared using the Krumhansl-Schmuckler key-finding algorithm (Krumhansl, 1990). This model is based on “key-profiles” of each potential key, which represent the stability of each pitch in the key, i.e., how well it fits in a tonal context (Krumhansl and Kessler, 1982). The pitch distribution of a given melody, weighted by duration, is compared to the key-profile of each key, and a correlation value is calculated. When correlations with the profiles of each potential key were calculated for each stem, the mean correlation with the correct key for AC stems [*r*(22) = 0.70] did not differ significantly from the mean correlation with the correct key of NC stems [*r*(22) = 0.73], *t*(44) = 1.24, *p* = 0.22 (averaging and statistics were performed on Fisher transformed correlation coefficients). The two groups of stems therefore did not differ in the degree to which they projected a sense of key.

### Procedure

Stimuli were played to participants over Logitech Z200 computer speakers at a comfortable listening volume within a sound attenuated room. The experiment was presented using PsychoPy (v1.79.01) on a MacBook Pro laptop, and sung responses were recorded as .wav files using the computer’s built-in microphone.

Each participant was instructed that s/he would hear the beginnings of some unfamiliar melodies and would need to “sing the note you think comes next.” Participants were asked to *continue* the melody—not necessarily complete it—on the syllable “la.” Each trial began when the participant pressed a button to hear a melodic stem. Immediately after the end of the last note of each stem, the word “Sing” appeared on the screen and participants were given 5 s to sing the continuation, after which they rated their confidence in their response on a 7-point Likert scale (1 = *low*, 7 = *high*).

Each participant was presented with 24 AC and 24 NC melodic stems (only one version from each AC-NC pair) in one of eight randomized presentation orders. (Three pairs were removed from analysis due to differences in the melodic contours of the two stems, hence data from 45 pairs was analyzed.) At the beginning of the experiment, each participant completed a pitch-matching task in which they heard and were asked to sing back a series of individual tones (F4, A4, B3, G#4, A#3, D4, C#4, and E^♭^4 [corresponding to 349.2, 440.0, 246.9, 415.3, 233.1, 293.7, 277.2, 311.1 Hz, respectively]; one octave lower for male participants). This was used to evaluate participants’ singing accuracy. Before the experimental trials began, participants were familiarized with the experimental procedure with a block of practice items, which ranged from simple scales and familiar melodies to unfamiliar melodies.

### Data Analysis

We extracted the mean fundamental frequency of the sung note using Praat (Boersma, 2002). The pitch of the sung note was determined by rounding the measured mean fundamental frequency to the closest semitone in the Western chromatic scale (e.g., A4 = 440 Hz), with the deviation from the frequency of this chromatic scale tone recorded (in cents, i.e., in hundredths of a semitone). The sung response was also represented in terms of its scale degree within the key of the stem in question. Responses were generalized across octaves for the purpose of this study. Participants’ responses to the pitch-matching portion of the experiment were also analyzed; if any participant’s pitch-matching responses did not round to the same note that was presented, or if their responses to at least 25% of the experimental trials were more than 40 cents away from the nearest semitone, the participant’s responses were excluded from further analysis (eight participants were omitted for these reasons). Additionally, reaction times were measured using a sound onset measurement script in Praat (a sound’s onset was detected when the sound reached a level -25 dB below its maximum intensity for a minimum of 50 ms) to determine how quickly the continuation was sung after the offset of the last note of the stem.

## Results

Participants found the task intuitive and uncomplicated, suggesting that the melodic cloze probability task provides a naturalistic way to measure melodic expectations. On average, participants sang a continuation note with a reaction time of 899 ms (*SD* = 604 ms), and their sung notes were an average of 1896 ms long (*SD* = 808 ms). Given that that the melodies had a tempo of 120 BPM, this corresponds to an average time interval of 1.80 beats after the offset of the stem, and a sung note duration of 3.79 beats.

### Constraint

**FIGURE 2:**
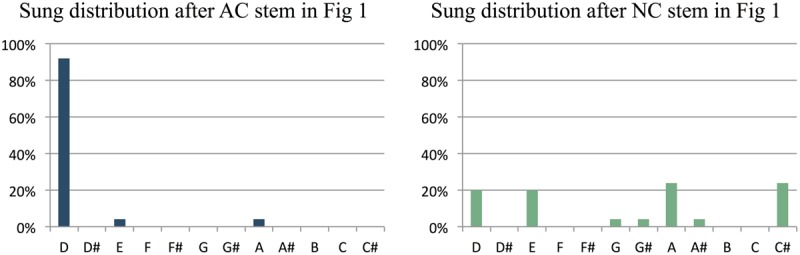
**Histograms showing the relative frequency of different notes sung by participants at the end of the AC and NC stems in Figure 1.** After the AC stem, most participants (92%) sang the pitch D, which is the 1st scale degree or tonic of the prevailing key of D major. After the NC stem, the note sung varied much more between participants: only 20% sang the pitch D, and no more than 24% of participants sang the same note (a tie between A and C# in this case).

The primary dependent variable in our study was the predictive constraint of a melodic stem, as measured by the percentage of participants that sang the most common note after the stem. **Figure 2** illustrates how this was computed, based on the AC-NC melodic pair in **Figure 1**. **Figures 2A,B** show the distributions of sung notes after the AC and NC stems in **Figure 1**, respectively. **Figure 2A** shows that 92% of participants that heard the AC stem produced the most commonly sung note (the tonic, D), while **Figure 2B** shows that no more than 24% of participants that heard the NC stem produced any one note (in this case, there was a tie between C# and A, but in most cases, one pitch class was most common). Thus the constraint of this melodic pair was 92% (or 0.92) for the AC melody and 24% (or 0.24) for the NC melody. For this pair, the AC melody was indeed far more constraining than the NC melody, as predicted.

**FIGURE 3:**
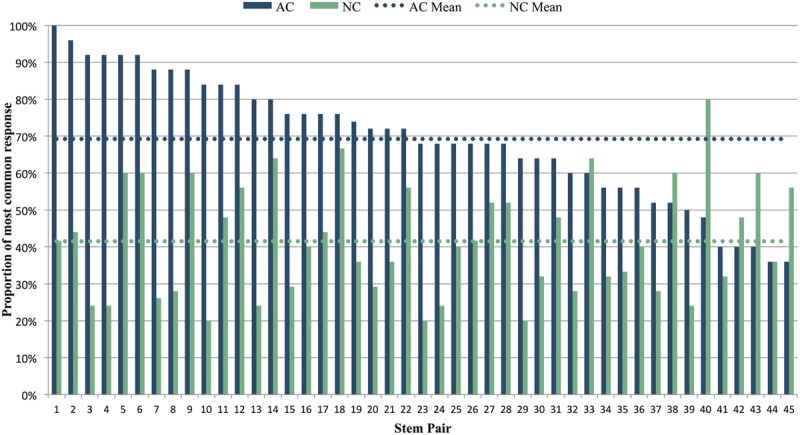
**Constraint of AC and NC stems, as calculated by the percentage of participants providing the most common response for each stem.** Stem pairs are ranked in order of decreasing constraint for AC stems. The melodic pair shown in **Figure 1** corresponds to stem pair 3 in this graph. Dotted horizontal lines show the mean constraint across all AC and NC stems.

For each AC and NC stem, we computed the constraint as described above. After AC stems, the average constraint was 69% (i.e., on average, 69% of participants sang the same note after hearing an AC stem), while after NC stems, the average constraint was 42% (i.e., on average, only 42% of participants sang the same note after hearing an NC stem). Thus on average, melodic stems in the AC condition did prove to be more constraining than NC stems (AC *M* = 0.692, *SD* = 0.171; NC *M* = 0.415, *SD* = 0.153), [*t*(44) = 7.79, *p* < 0.001]. This pattern of higher constraint for the AC vs. NC stem was observed in 38 of the 45 item pairs (**Figure 3**).

On average, participants responded significantly more quickly after AC stems (mean *RT* = 767 ms, *SD* = 265 ms) than after NC stems (mean *RT* = 1033 ms, *SD* = 302 ms), *t*(49) = 9.78, *p* < 0.001. Additionally, on average participants were significantly more confident in their responses to AC stems (*M* = 5.14, *SD* = 0.95) than to NC stems (*M* = 4.36, *SD* = 1.04), *t*(49) = 9.60, *p* < 0.001.

### Scale Degree

**FIGURE 4:**
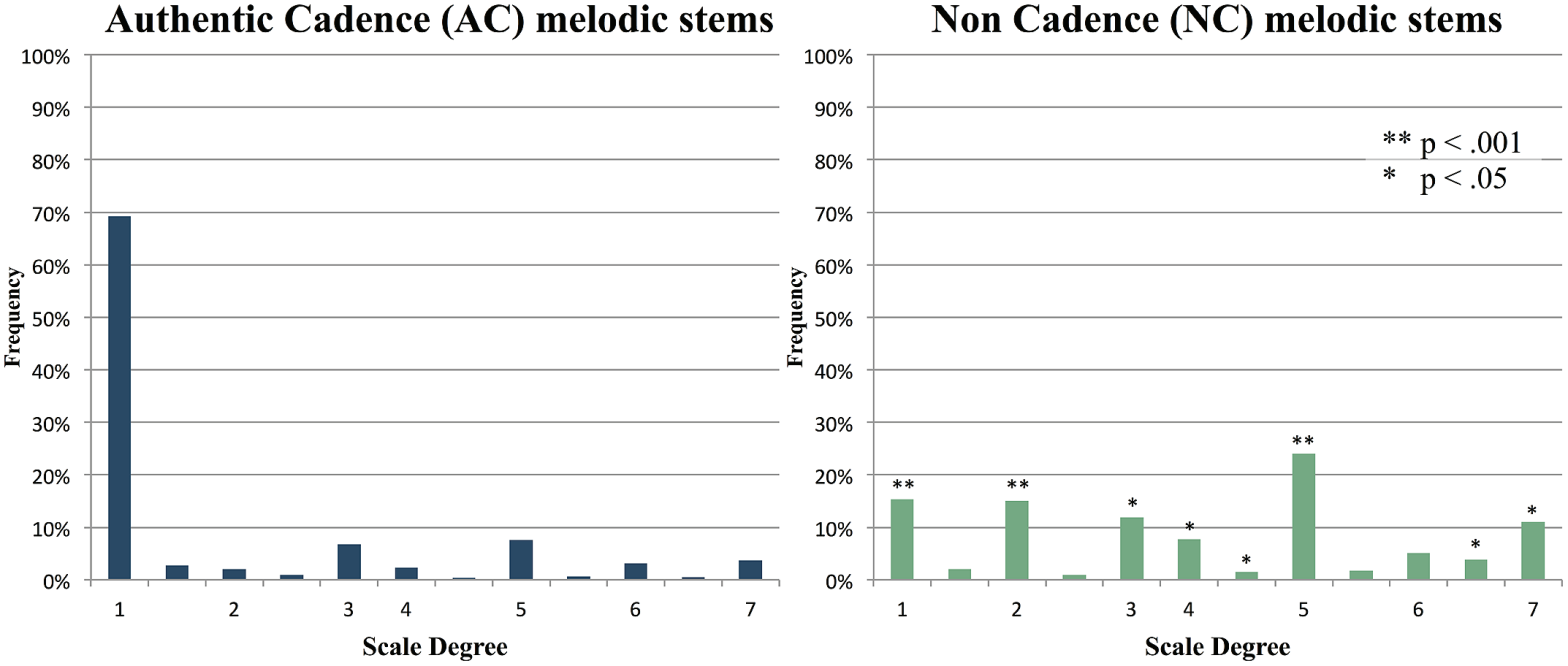
**Average of all response distributions to AC and NC stems, shown as scale degrees.** Numbers represent diatonic (major) scale degrees (e.g., 1 = tonic, 7 = leading tone, etc.), with asterisks indicating scale degrees with significantly different frequencies between the two conditions.

When responses were represented in terms of their scale degree in the key of the stem in question, and compiled across all items in each condition, the distributions for AC and NC items were strikingly different. For six of the seven diatonic scale degrees, the frequency of response differed significantly between AC and NC items based on *t*-tests of each scale degree with a Bonferroni correction applied (see **Figure 4** for *p*-values). For AC items, responses were heavily weighted around the first note of the scale, or tonic (known as ‘do’ in solfege). For NC items, responses were more widely distributed; however, they were mainly restricted to in-key diatonic scale degrees.

### Variability

**FIGURE 5:**
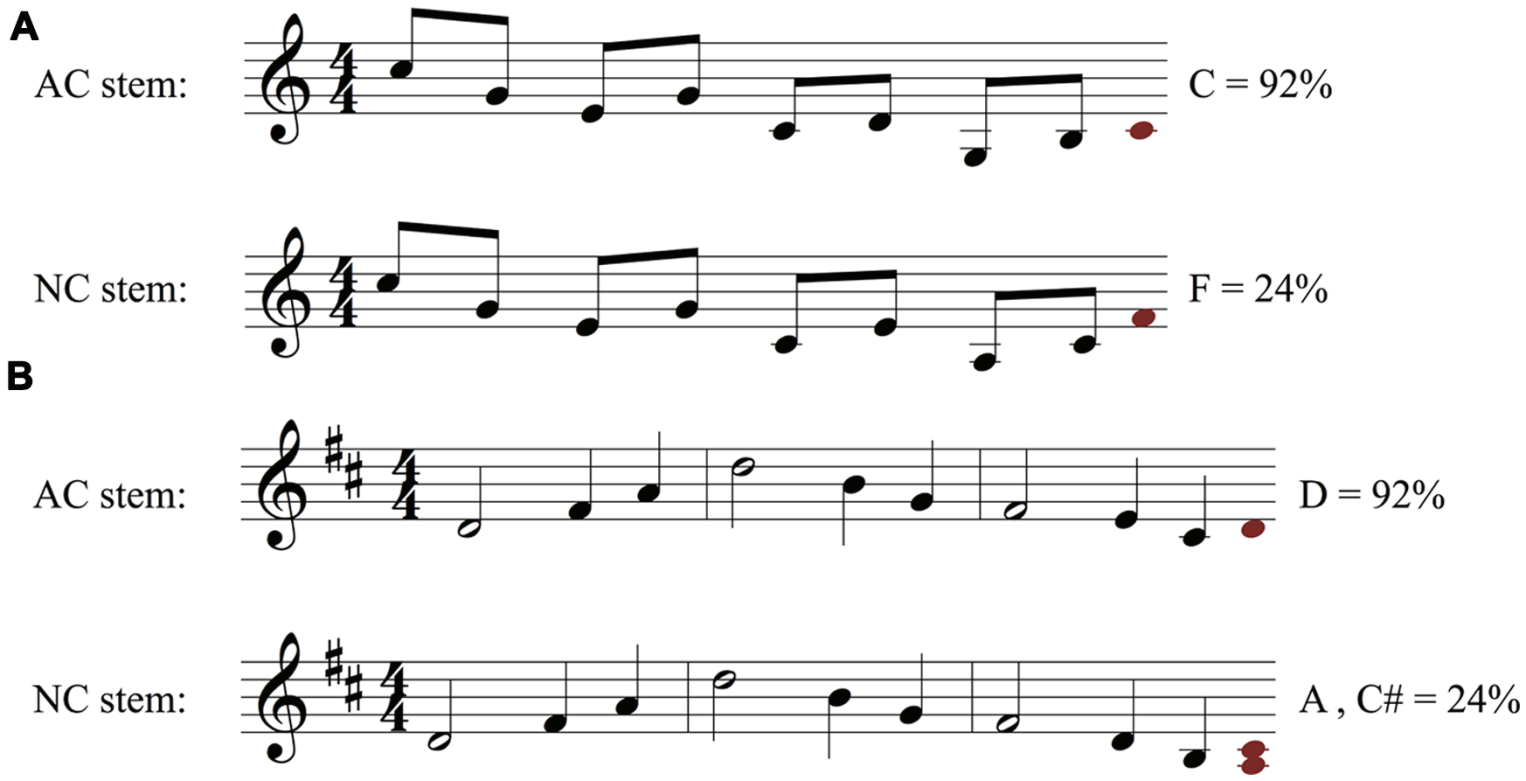
**Examples of two melodic stem pairs **(A,B)** with an AC stem that was much more constraining than the non-cadence (NC) stem.** Stems are shown in black and white, and for each stem the most frequently sung note is shown as a red note head at the end of the stem. The pitch class name of this note and the proportion of listeners who sang the note (i.e., the measured melodic constraint of the stem) are printed next to the red note. These two pairs correspond to stem pairs 4 and 3 in **Figure 3** [the stems in panel **(B)** are the same as in **Figure 1**].

**FIGURE 6:**
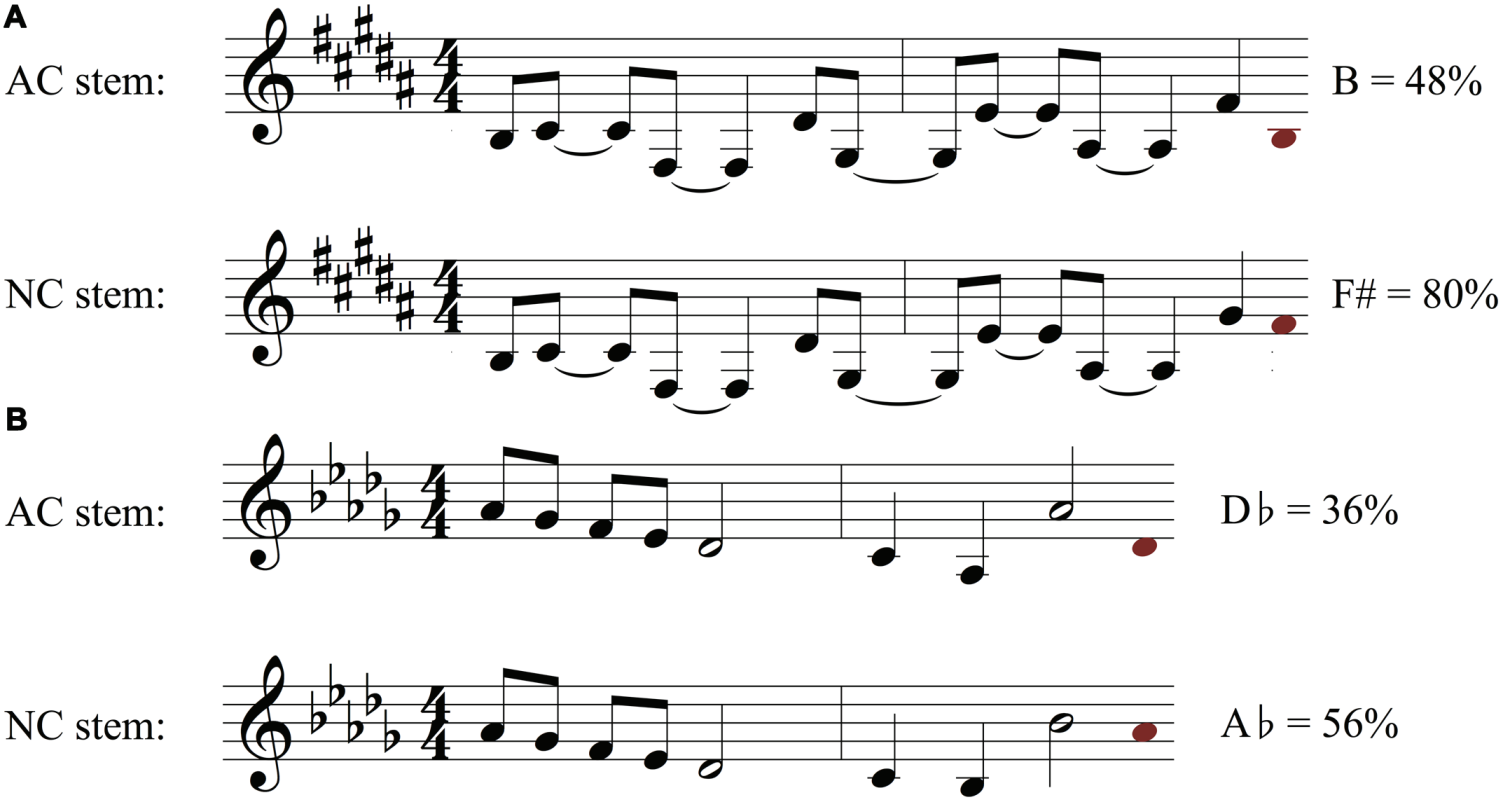
**Examples of two melodic stem pairs **(A,B)** with an AC stem that was *less* constraining than the NC stem.** Stems are shown in black and white, and for each stem the most frequently sung note is shown as a red note head at the end of the stem. The pitch class name of this note and the proportion of listeners who sang the note (i.e., the measured melodic constraint of the stem) are printed next to the red note. These two pairs correspond to stem pairs 40 and 45 in **Figure 3**.

While AC stems were on average significantly more constraining than their matched NC stems, there was considerable variability across AC-NC pairs in the degree of difference in constraint between members of a pair (see **Figure 3**). Thirty-eight out of 45 pairs demonstrated the expected pattern, with the AC stem proving more constraining than the NC stem. For instance, the stem pair in **Figure 5A** has a highly constraining AC stem, with 92% of participants singing the same note, the melody’s tonic pitch, C (in **Figures 5** and **6**, the most commonly sung note is shown as a red note head after the end of each stem). Why might this be? This stem is short, contains only one rhythmic value, and has very clear harmonic implications, beginning with an unambiguously arpeggiated tonic triad (C-E-G) and concluding with a similarly outlined complete dominant triad (G-B-D). This stem also ends on the leading tone of B, i.e., the seventh scale degree of the diatonic major scale, which customarily resolves to the tonic scale degree, particularly near the end of a phrase. Further structural factors that may contribute to the high degree of agreement on the final pitch are (1) the melody’s consistent downward contour, which seems to close in on middle C, and (2) the fact that the tonic note is heard very close to the end of the phrase, which may make it more likely to be replicated. Turning to the NC stem in **Figure 5B**, it is similar in many respects to the AC stem, yet very different in constraint, with the most commonly sung note (F) being produced by just 24% of participants who heard this stem. What might account for this? The NC stem does not have any resolution-demanding dominant pitches at its conclusion, and as a result lacks a clear sense of harmonic direction. Instead, the melody follows a downward pattern of melodic thirds (E–G, C–E, A–C) whose continuation is ambiguous. The most commonly chosen completion of F could be explained as the next logical pitch in the chain of descending thirds, after A–C. Thus, when faced with a stem where harmonic direction is underdetermined, subjects may have recruited an alternative strategy of melodic pattern continuation.

Another example of an AC stem that proved to be highly constraining is shown in **Figure 5B** (same melodic pair as in **Figure 1**). As with the melody in **Figure 5A**, the AC stem begins on the tonic note and returns to it as the most expected continuation, with an overall melodic range that emphasizes the octave generated above the first scale degree. The melody’s interior arpeggiates two chords, first the tonic (D–F#-A) in measure 1, then the subdominant (G-B-D) in measure 2. The subdominant chord frequently serves a syntactic role of “predominant,” a harmonic function that signals the initiation of a cadence. This is indeed how measure three is structured, with a heavily implied dominant harmony via scale degrees 2 and 7, and a melodic contour that insures D as a plausible completion due to an implied F#-E-D melodic descent and a unresolved leading tone of C#. The less constraining NC stem in **Figure 5B**, by contrast, ends on the sixth scale degree (the submediant). Unlike the leading tone, this note lacks a strong tendency to resolve in a particular way. It may plausibly serve as part of a stepwise motion to or away from the dominant, or as part of an arpeggiation of a predominant harmony; in either case, it negates the cadential function of the third measure and points to no obvious melodic completion.

Contrasting with these stems, where subjects’ responses to stems adhered to the AC/NC designations, there were several items where the constraint of the NC stem unexpectedly *exceeded* that of the AC stem. For example, after the NC stem in **Figure 6A**, 80% of participants sang the same note (F#, the 5th scale degree). In this particular melody, we believe this reflects the tendency for a large melodic interval to be followed by stepwise motion in the opposite direction. This “gap-fill” pattern (Meyer, 1956; Narmour, 1990) likely strongly influenced the continuation most participants chose, which involved singing a note (F#) one step down from the last note of the stem (G#), following a large leap of a sixth to an already contextually unstable note (scale degree six). Additionally, this stem has a strongly implied compound melody, wherein most of the topmost notes form a rising, stepwise pattern of B-C#-D#-E, which leads to an F# if this pattern is continued. Meanwhile, the unexpectedly low constraint of the AC stem in **Figure 6A** was perhaps due to the lack of a strong tendency note (like the leading tone) as its last pitch, and the obscuring of the underlying harmonic implications by the relative rhythmic complexity of the melody. That is, the unpredictable and syncopated rhythm may have reduced the strength of the expectancy for the tonic scale degree (Schmuckler and Boltz, 1994). Similarly, in the stem pair in **Figure 6B**, the most common continuation for the NC stem was a gap-filling motion to fill the exceptionally wide upward leap of an octave from Bb4–Bb5. Landing on Ab, which 56% of subjects agreed on, helps close that gap with a downward step and continues the melody on the more stable pitch of scale degree 5. This note also has the advantage of mirroring the first note of the melody, thus promoting melodic symmetry. The AC stem of this melodic pair presented no such clearly determined ending. If subjects opted to fill in the large upward octave gap to Ab with a downward step, they would land on the unstable fourth scale degree (Gb). On the other hand, if they were to resolve the melody with a cadence on the tonic note (Db), they would land far from the final note of the stem, going against a general tendency in melodic expectation for pitches that are proximate in frequency to the previous note (see section on modeling below).

Based on the above observations, it is clear that underlying harmonic structure, which was manipulated in the AC vs. NC stems, does not alone determine melodic expectation. Melodic factors that likely contributed to increased constraint in our melodies include (but are not limited to) rhythmic simplicity, gap-fill pattern, compound-line implication, leading-tone resolution, and pattern completion. In this way, stems in which linear, contrapuntal, rhythmic and harmonic parameters were closely coordinated produced reliable agreement on melodic completions, while examples with a conflict or ambiguity between those factors were prone to considerably less consensus.

### Musical Experience

**FIGURE 7:**
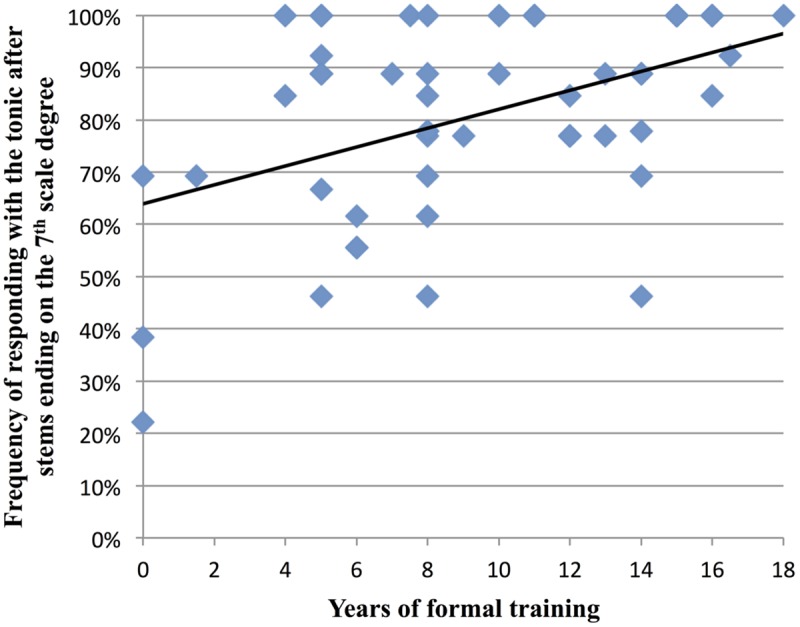
**Relationship between participants’ years of formal musical training and how often they sung the tonic after melodic stems that ended on the 7th scale degree.** On average, participants sang the tonic 81% of the time after these stems (data for all 50 participants are shown: due to some data points lying directly on top of each other, fewer than 50 data points are visible on the graph).

Prior research suggests that musical training enhances sensitivity to underlying harmonic structure (Koelsch et al., 2002). Since implicit harmony was used to guide the listeners’ expectation for a tonic note after AC stems, we sought to determine if participants with greater degrees of musical training were more likely to sing the tonic after AC stems. Thus across AC stems, we correlated each participant’s total years of formal musical training with their frequency of responding with the tonic. (Thus for example, if a participant sang the tonic after half of the AC stems they heard, their frequency of responding with the tonic to an AC stem would be 0.5.) When all AC items were included in the analysis, there was no significant correlation with years of formal musical training, *r*(48) = 0.035, *p* = 0.812. However, when we divided AC stems according to the scale degree of their final note, an interesting pattern emerged. On average, after AC stems that ended on the 7th scale degree, participants sang the tonic 81% of the time, and in these melodies, there was a significant correlation between participants’ years of formal training and their frequency of responding with the tonic, *r*(48) = 0.45, *p* = 0.001 (see **Figure 7**). This relationship with musical training was also observed with AC stems that ended on the 5th scale degree, where participants sang the tonic 55% of the time on average, *r*(48) = 0.33, *p* = 0.02. (The relationship was not seen for AC stems that ended on the 2nd scale degree, where participants sang the tonic 57% of the time on average.)

### Model Comparison

One potential use of the melodic cloze probability task is to test models of melodic expectation. While different forms of musical expectancy (e.g., melodic, rhythmic, harmonic) have been the subject of many important theoretical and empirical investigations (e.g., Schmuckler, 1989; Narmour, 1990; Schellenberg, 1996; Krumhansl et al., 1999; Large and Jones, 1999; Huron, 2006), melodic expectancy in particular has been a focus for quantitative modeling (e.g., Schellenberg, 1996; Krumhansl et al., 1999; Pearce et al., 2010). While comparison of behavioral and modeling data is not the primary focus of this paper, we present one such comparison to illustrate how melodic cloze data can be used for this purpose. We focus on the simplified version of the implication-realization (I-R) model of melodic expectancy (Narmour, 1990) developed by Schellenberg (1997).

This model computes the probability of each possible continuation of a melody based on two factors. The first of these factors is “pitch proximity,” which states that listeners expect the next tone of a melody to be proximate in pitch to the last tone heard. (Another way of stating this is that listeners generally expect melodies to move by small steps.) The second factor is “pitch reversal,” which states that after a leap, listeners expect the next tone to reverse direction (e.g., after an upward leap, they expect a downward pitch interval), and also expect the upcoming tone to land in a pitch region proximate to the penultimate tone (the first tone of the leap). A third factor relating to tonal stability was also included, based on values from the probe-tone profiles of Krumhansl and Kessler (1982). This factor reflects expectation for notes that fit well into the existing key context, with higher values for structurally more important/stable notes in key. Based on the equations for the simplified I-R model as codified in Schellenberg (1997) these three factors (proximity, reversal, and tonality) were weighted evenly by equalizing their maximum values, and were used to compute expectancies for all notes within two octaves of the final note of each melodic stem, using the MIDI toolbox (Eerola and Toiviainen, 2004).

**FIGURE 8:**
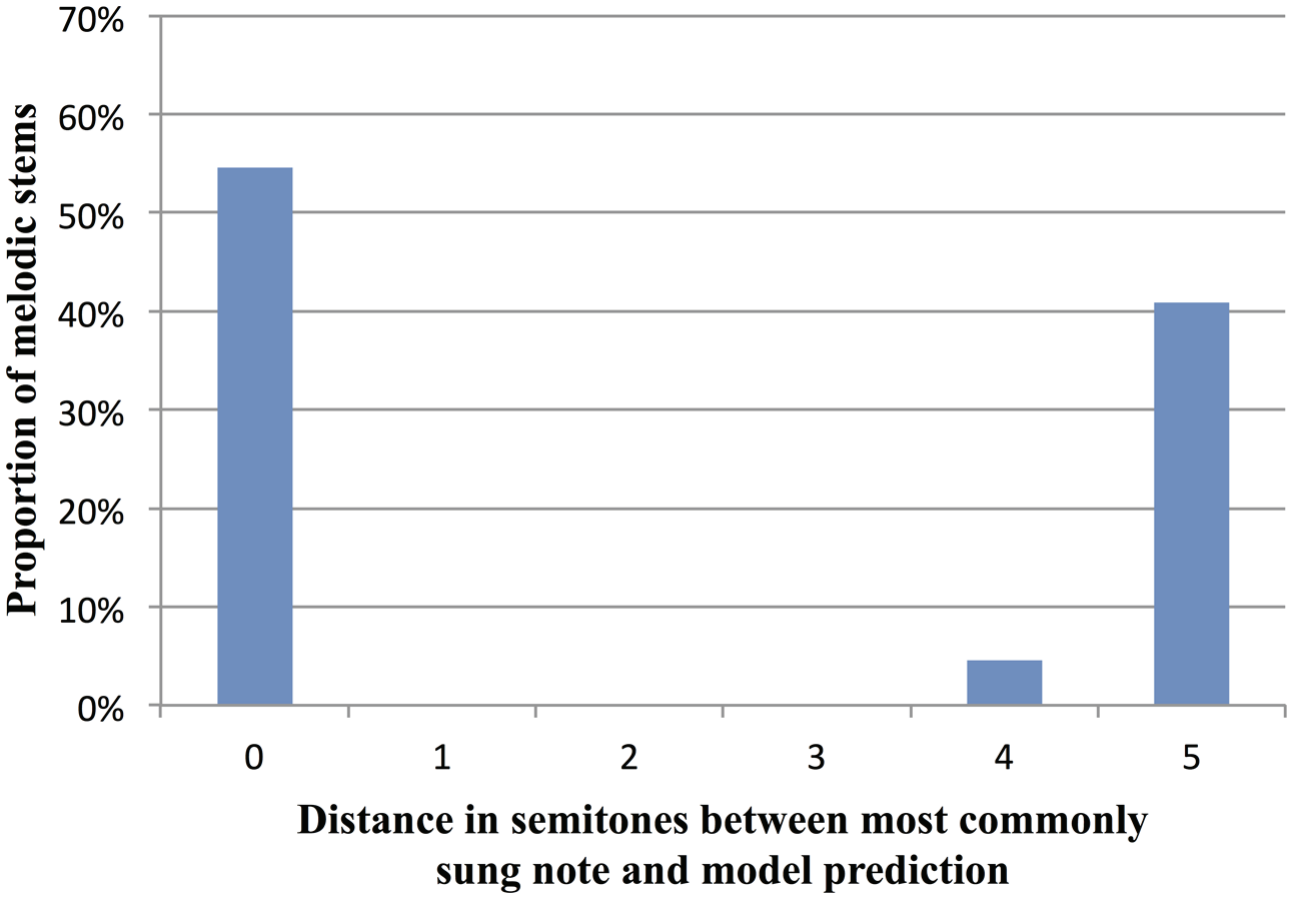
**Distance in semitones between the continuations sung by participants and the most likely continuation predicted by the simplified Implication-Realization (I-R) model of melodic expectancy (Schellenberg, 1997), including a tonality factor.** Data are for the 22 AC stems with a constraint of at least 69% (the AC average).

In order to compare the model’s predictions to cases where humans had strong expectations, we focused on high-constraint stems where most participants sang the same continuation (stems with constraint >69%, the mean of all AC stems). In the 22 stems satisfying this criterion, the simplified I-R model (including the tonality factor) correctly predicted the note most often sung by participants in 12 stems, i.e., 54.5% of the time. In the remaining 10 of these high-constraint stems (i.e., 45.5% of the time), the model’s predictions were an average of 4.9 semitones away from participants’ sung note (*SD* = 0.32 st) (see **Figure 8** for the distribution of distances between human data and model predictions). For the 10 stems where the model’s predictions differed from the mostly commonly sung note, we checked if the note predicted by the model was the *second*-most-commonly produced note by participants. This was true in only one stem. Overall, the model’s performance suggests that our data cannot be accounted for solely by local factors of proximity and reversal, combined with tonality. This suggests that larger-scale factors need to be taken into account, as further discussed below.

## Discussion

We introduce the melodic cloze probability task, in which participants hear the opening of a short, novel tonal melody and sing the note they expect to come next. This task, which is modeled on the well-known cloze probability task in psycholinguistics, has not previously been used to study expectancy in the field of music cognition. Participants found the melodic cloze task easy to do, demonstrating that expectancy can be measured in a comparable way across linguistic and musical domains.

Prior work using singing to study melodic expectancy has focused on responses to two-note intervals (see introduction for references). Of these studies, the closest task to ours is Lake (1987), who had participants sing extended continuations in response to a two-note interval preceded by a tonal context. Unlike the current study, the tonal context was not the opening of a novel coherent melody, but a sequence of notes consisting of a major chord, a scale, and another major chord, which served to establish a strong sense of key before the two-note interval. One might ask how our results compare to those of Lake, since one can conceive of our stimuli as also consisting of a key-inducing context followed by a final two-tone interval (i.e., the final two tones of the melodic stem).

**FIGURE 9:**
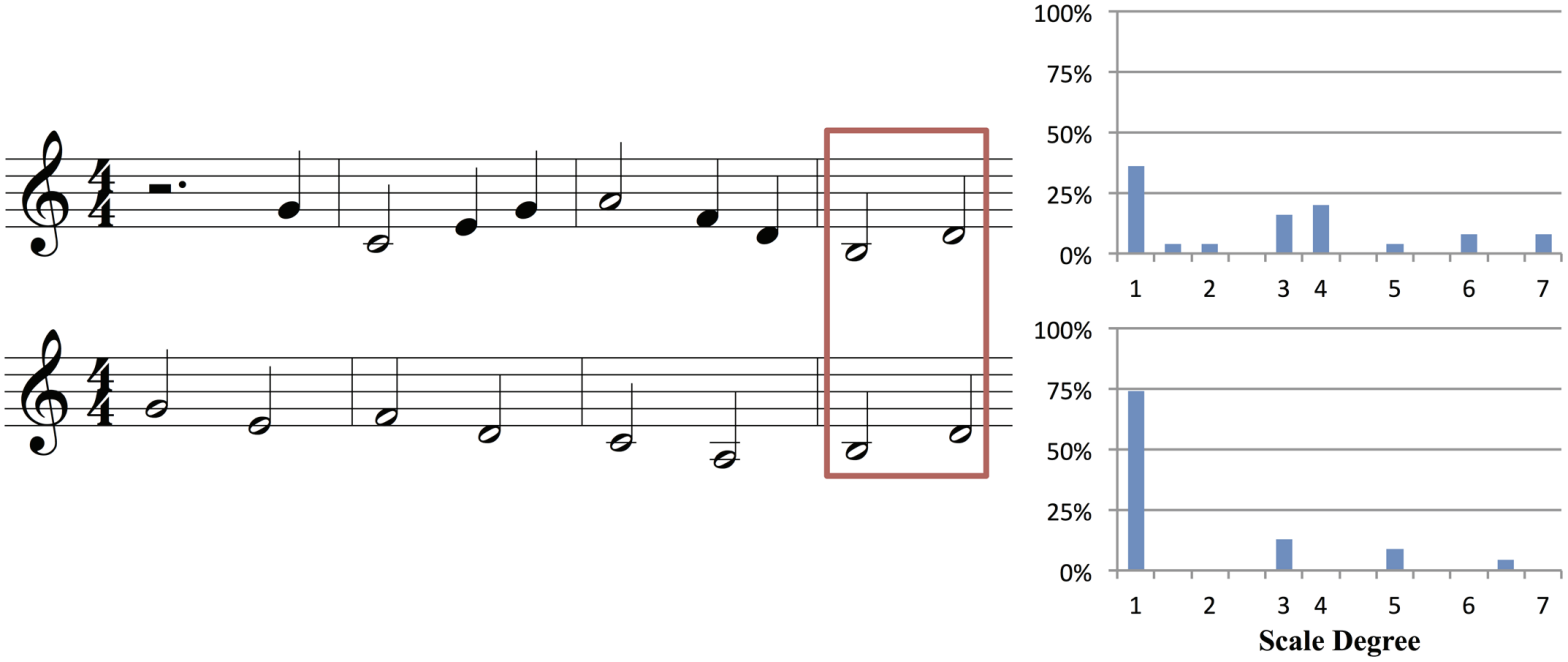
**Example of stems that have the same final two notes but elicit different patterns of responses from participants.** Stems have been transposed from their original keys to C major in order to facilitate comparison. Both stems end with scale degrees 7 and 2. The distribution of sung responses (expressed as scale degrees) is shown to the right of each stem.

While the last two notes of our stems clearly contribute to our results, our findings cannot be attributed to only hearing this final interval in a generic tonal context. A number of our stems are identical in the scale degrees of their final two notes, yet they elicit very different patterns of results from participants (see **Figure 9** for an example). This different pattern of responding to the same final interval reflects differences in the *structure* of the preceding notes. Thus our paradigm and results are not simply a replication of Lake (1987), and show the relevance of using melodically coherent materials as contexts for production-based studies of melodic expectation. Similarly, we note that our results are not simply a replication of the well-known probe-tone results of Krumhansl and Kessler (1982), since the pattern of responding was not just a reflection of the tonal hierarchy, and depended on the structure of the heard melody (e.g., **Figures 1** and **2**).

In addition to being the first study to obtain cloze probabilities for musical notes, to our knowledge the current study is the also the first to manipulate the predictive *constraint* of musical sequences as part of research on melodic expectation. By using pairs of monophonic melodic openings (or ‘stems’) matched in length, rhythm, and melodic contour, but differing in implied harmonic structure, we show that underlying harmonic progressions can strongly guide melodic expectations. Specifically, there was significantly more consistency in participants’ responses to melodic stems ending on an implied authentic cadence (AC condition) than in their responses to stems ending non-cadentially (NC condition), as reflected by a higher percentage of participants singing the most common continuation for items in the AC condition. In other words, AC stems were more highly constraining than NC stems on average.

However, our data also clearly indicate that expectations based on larger-scale implied harmony interact with expectations based on melodic structure. That is, despite the fact that the harmonic differences between the AC and NC melodies in each pair were similar, we observed considerable variability in the constraint of melodies. In some pairs, the AC stem was considerably more constraining than the NC stem, but in other pairs the difference in constraint was mild, and in seven pairs the NC stem was actually equal to or more constraining than the AC stem (**Figure 3**). Analysis of two such ‘reversed constraint’ pairs (**Figure 6**) suggested that factors related to rhythmic simplicity, gap-fill pattern, compound line implication, and pattern completion may have been involved in overwhelming harmonic expectations. Further investigation of the factors driving the observed large variation in constraint among melodies is clearly warranted. From our results it is clear that expectancies related to melodic patterns (e.g., gap-fill) may sometimes trump those related to tonality.

Indeed, the variability in constraint observed in our data (**Figure 3**) suggests that the melodic cloze task is well suited for use in future studies aimed at exploring the relative contributions of melodic and harmonic patterns in shaping melodic expectation. Such studies can help test and improve quantitative models of melodic expectation (e.g., Schellenberg, 1996, 1997; Krumhansl et al., 1999; Eerola and Toiviainen, 2004; Margulis, 2005; Pearce, 2005; Pearce and Wiggins, 2006). In the current study, we compared human melodic expectations to predictions based on Schellenberg’s (1997) simplified version of Narmour’s (1990) Implication-Realization (I-R) model of melodic expectation, with an added tonality factor. For the 22 AC melodies with a high degree of measured constraint (i.e., where >69% of participants sang the same note), the model correctly predicted the sung pitch in 54.5% of these melodies. In the remaining 45.5% of these melodies, the model predicted a pitch that was on average 4.9 semitones from the pitch actually sung by participants. This discrepancy between human expectations and model predictions likely stems from the fact that the simplified I-R model focuses on just the last interval of a melody, and does not take larger-scale structural patterns into account (such as harmonic progressions and recurring motivic patterns). Successful models of melodic expectation will almost certainly need to operate at multiple timescales, reflecting the human tendency to integrate both local and global information in processing melodic sequence structure (Dowling, 2010). In the future, it will be interesting to use the melodic cloze method to test models which are sensitive to patterns at multiple timescales, including Margulis’ (2005) model of melodic expectation, and Pearce’s (2005) IDyOM model (cf. Pearce and Wiggins, 2006). Such models can be tested and improved by comparing their predictions with observed cloze probabilities from human participants.

The musical cloze probability task has further uses in the field of music cognition. For example, this paradigm can be used to investigate how different factors influence melodic expectancy. While we manipulated only the harmonic structure of melodies in the present experiment, the influence of any other factor (e.g., melodic contour, rhythm, dynamics, etc.) on musical expectations could be explored in subsequent studies by composing melodies in pairs and manipulating the one factor while keeping other factors constant. Additionally, the task could be varied to have participants sing multiple-note continuations, as has been done in previous studies (Carlsen, 1981; Lake, 1987; Unyk and Carlsen, 1987; Thompson et al., 1997; Schellenberg et al., 2002). This would allow responses to be examined on longer timescales than just the first sung note. In addition, it would reduce the possibility that participants are responding by *completing* the melodic sequences with the sung note, instead of *continuing* them (as instructed). This is an important issue, as the note sung after the stem may differ depending on whether listeners treat it as a continuation or a completion (Aarden, 2003, cf. Huron, 2006).

Of course, the melodic cloze paradigm does have its limitations. By focusing on what pitch a person sings, it cannot give independent measures of all the different types of expectations which may be at play at a given point in a melody, such as timbral expectations (if listening to complex textures) or rhythmic expectations. To study these sorts of expectations, modifications of the paradigm presented here would be necessary. For example, if studying rhythmic expectations, at the end of each stem one could ask participants to press a bar for as long as they think the next note will last.

The melodic cloze task can also be used to examine musical expectations in different populations. We observed a significant correlation between formal musical training and a tendency to sing the tonic after AC stems that ended on the 7th or 5th scale degrees. It has been suggested that having more musical experience leads to greater sensitivity to harmonic cues, which is consistent with our finding and with neural research on harmonic processing (Koelsch et al., 2002). Future studies could use the melodic cloze method to investigate how different kinds of musical experience might impact expectancy formation. For example, expectations may differ between musicians who have been educated in music theory vs. those who have experience singing or improvising without reading music. Additionally, the melodic cloze paradigm could be used in studies with children, to investigate how melodic expectations develop (cf. Corrigall and Trainor, 2014).

Obtaining melodic cloze probabilities is crucial for future research comparing predictive processing in music and language, as it allows for the comparison of the effects of violating predictions of comparable strength in the two domains (cf. Tillmann and Bigand, 2015). Previous studies comparing expectancy violations in music and language have typically chosen violations that are intuitively thought to be comparable in the two domains. By using a cloze paradigm to quantify cloze probabilities for possible continuations in both domains, it is possible to compare effects of violations of the same degree, using normed stimuli (cf. Featherstone et al., 2012). For example, this will allow comparison of brain responses to plausible violations of expectations, instead of to frank structural violations (which rarely occur in naturalistic sequences). Also, studies that probe interactions between simultaneously presented music and language expectancy violations can be more precisely calibrated, in order to further elucidate cognitive and neural relations between language and music processing.

## Conflict of Interest Statement

The authors declare that the research was conducted in the absence of any commercial or financial relationships that could be construed as a potential conflict of interest.

## Footnotes

1 Waters et al. (1998) used what they refer to as a “musical ‘cloze’ task,” but theirs was a multiple-choice task where participants selected one of several pre-composed sections of musical notation.
